# All-trans Retinoic Acid-induced Abnormal Hippocampal Expression of Synaptic Genes *SynDIG1* and *DLG2* is Correlated with Anxiety or Depression-Like Behavior in Mice

**DOI:** 10.3390/ijms21082677

**Published:** 2020-04-11

**Authors:** Xin-Ya Qin, Hui Fang, Qing-Hong Shan, Cong-Cong Qi, Jiang-Ning Zhou

**Affiliations:** 1 Chinese Academy of Sciences Key Laboratory of Brain Function and Diseases, School of Life Sciences, University of Science and Technology of China, Hefei 230027, China; qinxinya@ustc.edu.cn (X.-Y.Q.); fanghui@ustc.edu.cn (H.F.); shanqh@ustc.edu.cn (Q.-H.S.); 2 Department of Laboratory Animal Science, Fudan University, Shanghai 200000, China; qcc@mail.ustc.edu.cn

**Keywords:** all-trans retinoic acid, discs large homolog 2, synapse differentiation-inducing gene protein 1, hippocampus, anxiety-like behavior, depression-like behavior

## Abstract

Clinical reports suggest a potential link between excess retinoids and development of depression. Although it has been shown that all-trans retinoic acid (ATRA) administration induces behavioral changes, further insight into how ATRA is involved is lacking. The hippocampus seems to be a major target of retinoids, and abnormal synaptic plasticity of the hippocampus is involved in depression. We examined two genes associated with synaptic function, discs large homolog 2 (*DLG2*), and synapse differentiation-inducing gene protein 1 (*SynDIG1*) in terms of hippocampal expression and correlation with behavior. Three different doses of ATRA were injected into young mice and 10 mg/kg ATRA was found to induce depression-like behavior. In the hippocampus, *DLG2* mRNA was significantly decreased by ATRA. mRNA levels were positively correlated with central area duration and distance in the open-field test. Increased *SynDIG1* mRNA levels were observed. There was a negative correlation between *SynDIG1* mRNA levels and mobility time in the forced swimming test. Retinoic acid receptor γ mRNA was significantly positively correlated with *DLG2* and negatively correlated with *SynDIG1*. To summarize, ATRA administration induced anxiety- and depression-like behavior accompanied by a decreased expression of *DLG2* and an increased expression of *SynDIG1*. Moreover, *DLG2* was correlated with anxiety-like behavior and *SynDIG1* was correlated with depression-like behavior. These results might constitute a novel target underlying ATRA-induced anxiety- and depression-like behavior.

## 1. Introduction

Retinoic signaling is reportedly linked with the development of the central nervous system (CNS) and the pathogenesis of depression in adults [1,2,3,4]. Excessive consumption of vitamin A (hypervitaminosis A) has long been known to cause adverse psychiatric events [5]. A synthetic retinoid used to treat acne, 13-*cis*-retinoic acid (13-*cis*-RA; isotretinoin), has been linked to depression and suicide, since its approval in 1982 [6]. All-trans retinoic acid (ATRA), the endogenous active derivative of vitamin A, plays a role in cell growth, neural differentiation, and synaptic plasticity during development and operates exclusively by regulating gene transcription [7,8,9]. Recently, our group found that chronic ATRA administration could induce depression-like behavioral changes in adult rats [10,11,12]. 

The hippocampus is involved in mood disorders, such as anxiety and depression. Brain imaging and post-mortem studies provide evidence of changes in the cellular architecture and/or morphology within this brain region, including a reduction in hippocampal volume, and atrophy of hippocampal pyramidal neurons [13,14,15,16]. Recent studies have indicated that abnormal synaptic plasticity in specific areas of the CNS, particularly the hippocampus, may be a core factor in the pathophysiology of depression [17]. Studies in rodents have provided evidence in support of a reduced synapse number and decreased levels of synaptic signaling proteins in the hippocampus in a depression model [18,19]. Furthermore, antidepressants, such as the highly prescribed selective serotonin reuptake inhibitors, could enhance synaptic plasticity in the hippocampus, as demonstrated in electrophysiological studies [20,21]. All of the above studies confirm a link between altered synaptic plasticity in the hippocampus and major depression. Moreover, the hippocampus seems to be a main target of retinoids [20]. Our group found that ATRA-induced impairments in hippocampal neurogenesis correlate with depression-like behavior [11]. It has been reported that ATRA treatment enhances excitatory synaptic transmission in the hippocampus [21,22] and ATRA is mediated in synaptic plasticity via a synaptic protein synthesis-dependent mechanism [23]. These reports demonstrate the potential role of ATRA in synaptic plasticity of the hippocampus. To better understand the molecular mechanism of synaptic plasticity as influenced by ATRA, we used a whole-genome complementary DNA microarray to investigate changes of gene expression in the human neuroblastoma cell line BE2(c) cells after ATRA administration. Several genes are altered by ATRA (Table S1). We only chose these two synaptic-associated genes, discs large homolog 2 (*DLG2*) and synapse differentiation-inducing gene protein 1 (*SynDIG1*), among the genes altered by ATRA. *DLG2* is thought to have vital roles in synaptic plasticity [24]. It has been reported that a reduction of *DLG2* expression is found in the hippocampus in depression disorders [25], and *DLG2* might be involved in depression disorders, according to results of a genome-wide association study [26]. *SynDIG1* has been identified as an α-amino-3-hydroxy-5-methylisoxazole-4-propionic acid subtype glutamate receptor (AMPAR)-interacting transmembrane protein that could regulate excitatory synapse development via AMPAR content [27]. Moreover, *SynDIG1* has been found to be involved in depressive symptoms in a genome-wide association study [28].

In this study, we aimed to investigate the effect of ATRA on the expression of two synaptic- associated genes, *DLG2* and *SynDIG1*, in the hippocampus and their relationship with anxiety- or depression-like behavior in young mice.

## 2. Results

### 2.1. ATRA-Induced Anxiety- and Depression-Like Behavior in Young Mice

**Figure 1:**
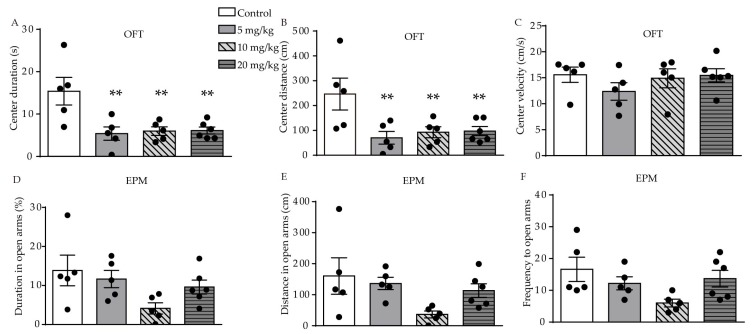
Effects of different doses of ATRA on anxiety-like behavior in mice. (**A**–**C**) center duration, distance and velocity in OFT in ATRA treatment and control groups; (**D**–**F**) duration, distance and frequency in open arms in EPM test. Data are expressed as mean ± SEM, with *n* = 5–6 in each group. ** *p* < 0.01 versus controls, using one way ANOVAs with least significant difference (LSD) test.

We used the open-field test (OFT), elevated-plus maze (EPM), forced swimming test (FST), and sucrose preference test (SPT) behavioral tests to investigate the effect of ATRA on anxiety- and depression-like behavior in young mice. In the OFT, significant difference in duration (F (3, 17) = 6.558, *p* = 0.0038, Figure 1A) and distance (F (3, 17) = 4.828, *p* = 0.0131, Figure 1B) traveled in the central area were found among the control, 5, 10, and 20 mg/kg ATRA groups. Post-hoc analysis revealed ATRA treatment significantly decreased the duration (*p* = 0.0016, *p* = 0.0025, *p* = 0.0020) and distance (*p* = 0.0036, *p* = 0.0090, *p* = 0.0083) traveled in the central area among the groups receiving 5, 10, and 20 mg/kg doses. There was no significant difference in the velocity of movement in the center area among the four groups in the OFT (F (3, 17) = 0.9254, *p* = 0.4497, Figure 1C). This indicates that ATRA did not affect motor function in mice, in comparison with the vehicle. The results of the EPM showed that there was no significant difference in the time (F (3, 17) = 2.713, *p* = 0.0773, Figure 1D), distance (F (3, 17) = 2.713, *p* = 0.0847, Figure 1E), and frequency (F (3, 17) = 2.808, *p* = 0.0709, Figure 1F) in the open arms. 

**Figure 2:**
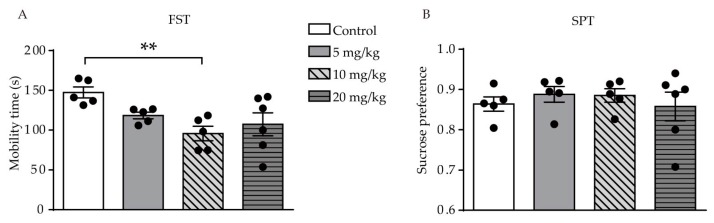
Effects of different doses of ATRA on depression-like behavior and anhedonia level in mice. (**A**) Mobility time in the FST. (**B**) result of the SPT in ATRA and control groups. Data are expressed as mean ± SEM, with *n* = 5–6 in each group. ** *p* < 0.01 versus controls, using one way ANOVAs with LSD test.

Results of the FST showed a difference was found among the four groups (F (3, 17) = 4.555, *p* = 0.0162, Figure 2A). The result showed that only mice who were administered a dose of 10 mg/kg ATRA had a significant reduction in mobility time during the 6-min period, as compared with the control group (*p* = 0.0027). There was no significant difference in mobility time during the FST between the 5 and 20 mg/kg groups and the control group (*p* = 0.0670, *p* = 0.0823, respectively). In the SPT, there was no significant difference between all ATRA groups and vehicle, which suggested that anhedonia was not affected by ATRA administration (F (3, 17) = 0.3580, *p* = 0.7840, Figure 2B). These results indicate that ATRA induced depression-like behavior in young mice.

### 2.2. ATRA-Induced Changes in mRNA Expression of DLG2, SynDIG1, and Retinoic Acid Receptors in the Hippocampus

**Figure 3:**
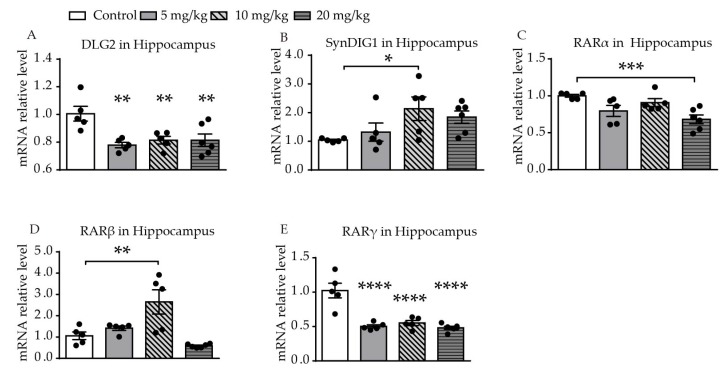
Effects of different doses of ATRA on mRNA expression levels of target genes in the hippocampus. Changes in mRNA expression levels of (**A**) *DLG2*, (**B**) *SynDIG1*, (**C**) *RARα*, (**D**) *RARβ*, and (**E**) *RARγ* in mice treated with 5 mg/kg, 10 mg/kg, and 20 mg/kg RA, compared with controls. Data are expressed as mean ± SEM, with *n* = 5–6 in each group. * *p* < 0.05, ** *p* < 0.01, *** *p* < 0.001, **** *p* < 0.0001 versus controls, using one way ANOVAs with LSD test.

Significantly different levels of *DLG2* mRNA expression were found among the four groups (F (3, 17) = 6.391, *p* = 0.0043, Figure 3A). Levels of expression of *DLG2* mRNA in the hippocampus were significantly decreased in all of the ATRA treatment groups, compared with the control group (*p* = 0.0011, *p* = 0.0042, *p* = 0.0030). A difference in expression of *SynDIG1* mRNA was found among the four groups (F (3, 17) = 3.108, *p* = 0.0469, Figure 3B). Expression of *SynDIG1* mRNA was significantly increased at a dose of 10 mg/kg ATRA (*p* = 0.0142). To investigate the possible relationship between the expression of synaptic-related genes and ATRA receptor, mRNA expression of three types of ATRA receptors in the hippocampus were simultaneously measured. A significant difference in retinoic acid receptor α (*RARα*) mRNA levels was found among the four groups (F (3, 17) = 6.252, *p* = 0.0047, Figure 3C). The 20 mg/kg ATRA treatment group showed a significant reduction in *RARα* mRNA levels (*p* = 0.0008). There was a significant difference in *RARβ* mRNA expression among the four groups (F (3, 17) = 9.459, *p* = 0.0007, Figure 3D). The expression of *RARβ* mRNA in the hippocampus was increased with administration of 10 mg/kg ATRA, compared with the control (*p* = 0.0014). Significantly different levels of *RARγ* mRNA expression were also found among the four groups (F (3, 17) = 20.54, *p* < 0.0001, Figure 3E). The expression of *RARγ* mRNA in all of the ATRA treatment groups was significantly decreased (*p* < 0.0001).

### 2.3. Association of DLG2 and SynDIG1 mRNA Levels with Anxiety- and Depression-Like Behavior and RARs in the Hippocampus

**Figure 4:**
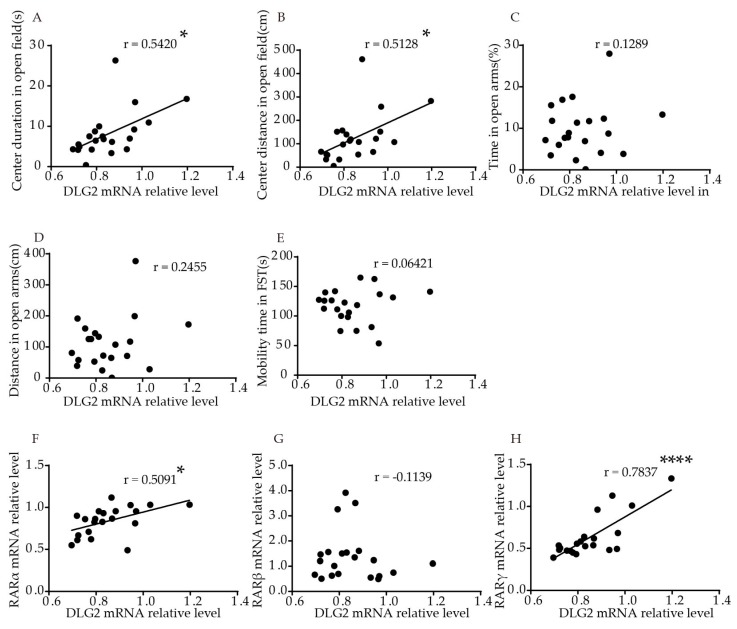
Correlation of *DLG2* mRNA levels in the hippocampus with behavior and *RARs* mRNA levels in young mice. Correlation between mRNA levels of *DLG2* in the hippocampus and (**A**) center duration, (**B**) center distance in OFT, (**C**) duration, and (**D**) distance in the open arms in EPM, (**E**) mobility time in FST. Correlation between mRNA levels of *DLG2* in the hippocampus and (**F**) *RARα*, (**G**) *RARβ,* and (**H**) *RARγ*. Correlation analysis was performed using Pearson’s correlation test. * *p* < 0.05, **** *p* < 0.0001.

We performed correlation analysis, to explore the association of *DLG2* and *SynDIG1* expression with anxiety- and depression-like behavior in mice. The results showed that relative *DLG2* mRNA levels in the hippocampus were significantly positively correlated with duration (*p* = 0.0111, r = 0.5420, Figure 4A) and distance (*p* = 0.0174, r = 0.5128, Figure 4B) traveled in the central area in the OFT. No significant correlation was found between *DLG2* with time (*p* = 0.5778, Figure 4C) and distance (*p* = 0.2834, Figure 4D) in the open arms in the EPM or mobility time in FST (*p* = 0.7821, Figure 4E). Table S2 shows the correlation more intuitively. Furthermore, relative *DLG2* mRNA levels in the hippocampus were significantly positively correlated with relative mRNA levels of *RARα* (r = 0.5091, *p* = 0.0184, Figure 4F) and *RARγ* (r = 0.7873, *p* < 0.0001, Figure 4H). No significant correlation was found between *DLG2* and *RARβ* mRNA (r = −0.1139, *p* = 0.6231, Figure 4G).

**Figure 5:**
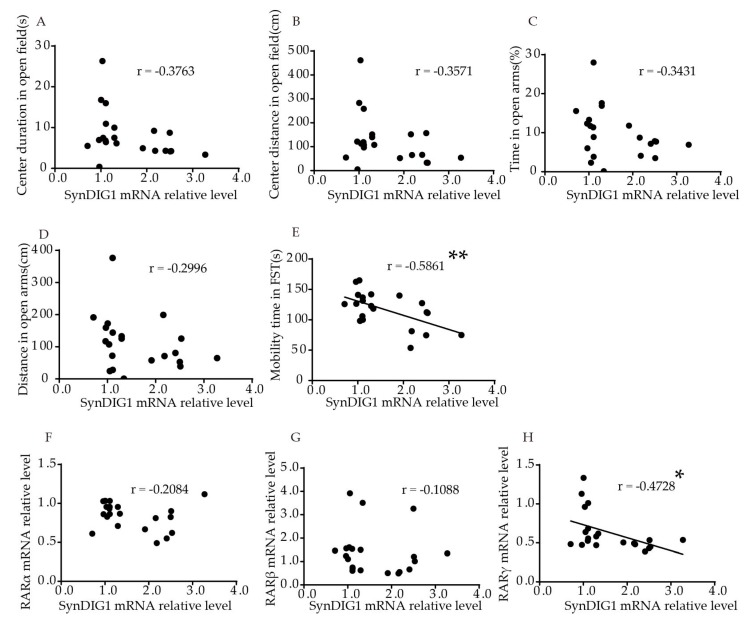
Correlation of the *SynDIG1* mRNA levels in the hippocampus with behavior and *RARs* mRNA levels in young mice. Correlation between mRNA levels of *SynDIG1* in the hippocampus and (**A**) center duration, (**B**) center distance in OFT, (**C**) duration, and (**D**) distance in the open arms in EPM, (**E**) mobility time in FST. Correlation between mRNA levels of *DLG2* in the hippocampus and (**F**) *RARα*, (**G**) *RARβ,* and (**H**) *RARγ*. Correlation analysis was performed using Pearson’s correlation test. * *p* < 0.05, ** *p* < 0.01.

Interestingly, relative *SynDIG1* mRNA levels in the hippocampus were significantly negatively correlated to mobility time in FST (*p* = 0.0052, r = −0.5861, Figure 5E). No significant correlation was found between relative SynDIG1 mRNA levels and duration (*p* = 0.0927, Figure 5A), or distance (*p* = 0.1120, Figure 5B) traveled in the central area in the OFT, or time (*p* = 0.1278, Figure 5C) and distance (*p* = 0.1871, Figure 5D) in open arms in EPM. Table S2 shows the correlation more intuitively. The results showed that relative *SynDIG1* mRNA levels in the hippocampus were significantly negatively correlated with *RARγ* (r = −0.4728, *p* = 0.0304, Figure 5H). No significant correlation was found between relative mRNA levels of *SynDIG1* and *RARα* (r = −0.2084, *p* = 0.3647, Figure 5F), or *RARβ* (r = –0.1088, *p* = 0.6387, Figure 5G).

## 3. Discussion

This study showed that short-term administration of ATRA at a dose of only 10 mg/kg induced depression-like behavior in young mice, accompanied by a decreased expression of *DLG2* and an increased expression of *SynDIG1*. In addition, *DLG2* in the hippocampus was correlated with anxiety-like behavior, and *SynDIG1* was correlated with depression-like behavior.

Clinical reports showing the onset of the depressive symptoms occurred after the use of Accutane (a 13-cis isomer of all-trans retinoic acid to treat severe cystic acne) in humans, predominantly in adolescents, among whom the rate of neurogenesis is predicted to be relatively high [2,3,29]. Modelling adolescence in animal models is contentious but in rodents young animals 4–6 weeks of age have been suggested to represent a post-weaning period of sexual maturation that is associated with rapid growth and reproduces some of the neurodevelopmental effects observed in human adolescence [30]. To parallel these conditions, our studies were performed in young mice. To avoid the trauma of daily oral gavage, ATRA was injected into the abdominal cavity. Previous articles also reported that administration of ATRA or 13-cis-RA increases depression-related behavior in young mice or rats [10,31]. According to the previous reports, 10 mg/kg retinoic acid administered for 14 days impaired the formation of a reward-induced positive bias in rodents, as seen in human depression [32,33,34]. We therefore explored the effect of 10 mg/kg in depression-like behaviors. We selected a higher dose of 20 mg/kg and a lower dose of 5 mg/kg within the range of previously reported doses [33,35]. In a previous study, exposure to 13-cis-RA (1 mg/kg daily, less than the concentration in our experiment) was extended to 21 days from 7 days, and a significant decrease in cell proliferation was apparent in the hippocampus [36]. It has been reported that chronic administration of 13-cis-RA (1 mg/kg) for 6 weeks induces depression-related behaviors in young mice [31]. Cai et al. reported that 6-week injection of ATRA (2 mg/kg) induced behavioral changes in young rats [10,37]. Hu et al. reported that a 19-day course of ATRA injected into the lateral cerebral ventricle induced typical depression-like behavior in adult rats [38]. These study findings are consistent with our results in the EPM and FST. Our experiments are the first to extensively examine the dose effect of short-term injection of ATRA on depression-like behavior in mice, with 10 mg/kg ATRA treatment for 12 days inducing depression-like behavior in young mice. However, administration of ATRA did not induce anhedonia, the main symptom of depression, in the SPT, which has also been shown in other experiments using ATRA and 13-cis-RA [10,39,40]. 

A previous report reviewed 1191 articles describing 532 genes regulated by ATRA [41], not including the two genes in our study. Our study was the first to identify a change in expression of the *DLG2* and *SynDIG1* genes by ATRA. *DLG2*, associated with excitatory synapse, is thought to have vital roles in synaptic plasticity and is involved in AMPA receptor trafficking and formation of N-methyl-D-aspartate receptor (NMDAR)-associated complexes, which has an effect on long-term potentiation in the hippocampus [24]. A previous study reported altered expression of synapse- related genes on post-mortem examination of individuals with major depressive disorder [42]. Furthermore, the reduction of *DLG2* mRNA expression in the hippocampus has been found in depression disorders [25], which is consistent with our results in an ATRA-induced model of depression. Additionally, correlation analysis showed that hippocampal *DLG2* mRNA levels had a closely positive correlation with duration and distance traveled in the central area in the OFT. These data showed that expression disturbance of *DLG2* was closely involved in anxiety-associated behaviors in the OFT. Thus, the specific role of *DLG2* in the pathogenesis of anxiety warrants further examination.

Surprisingly, *SynDIG1* was significantly increased by ATRA in the hippocampus of mice. It has been reported that *SynDIG1* regulates the number of functional excitatory synapses, altering both AMPA and NMDA receptor-mediated transmission [43]. A previous study reported increased basal glutamatergic transmission in the CA1 area of the hippocampus in a rat model of depression, in comparison with control rats [44]. Whether increased expression of *SynDIG1* might be associated with excitatory synaptic glutamatergic transmission in the hippocampus, which was paralleled by depression-like behavior induced by ATRA administration, needs further study. Moreover, we found a close correlation between hippocampal *SynDIG1* mRNA levels and mobility time in the FST. Increased *SynDIG1* may contribute to decreased mobility time in the FST. The strong correlation after ATRA exposure suggests that changes in *SynDIG1* mRNA levels were paralleled by and strongly linked to depressive symptoms; however, the specific role of *SynDIG1* in the pathogenesis of depression has yet to be determined.

The role of retinoic acid (RA) lies mainly in its binding to nuclear retinoid receptor proteins called retinoic acid receptors (*RARα*, *RARβ*, and *RARγ*) and retinoid “X” receptors (*RXRα*, *RXRβ*, and *RXRγ*) as transcription factors. *RARs* bind ATRA and 9-*cis*-RA with high affinity whereas *RXRs* exclusively bind 9-*cis*-RA [45]. We detected the expression of *RARs*. The findings of a previous report were consistent with our results, showing that *RARα* mRNA levels were decreased and *RARβ* was increased in the hippocampus modulated by ATRA [46]. Moreover, our results showed that *RARγ* mRNA levels were significantly positively correlated with *DLG2* and negatively correlated with *SynDIG1*. Our findings might provide insight into *RARγ* involvement in ATRA-mediated *DLG2* and *SynDIG1* expression.

## 4. Materials and Methods 

### 4.1. Animals and Drugs

**Figure 6:**
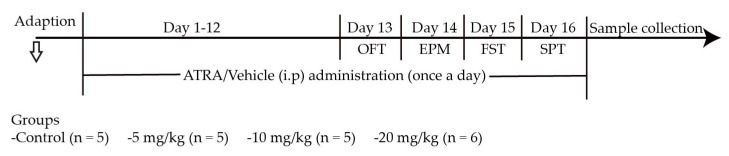
Experimental schedules for administration of ATRA and testing for anxiety- and depression-like behavior to explore the effect of ATRA on changes in behavior and synaptic gene expression in mice. OFT: open-field test; EPM: elevated-plus maze; FST: forced swimming test; SPT: sucrose preference test.

Three-week-old healthy male C57/BL6 mice were grouped and housed in cages (five per cage) with free access to food and water and were maintained on a 12-h light–dark cycle (lights on, 8:00 am; lights off, 20:00 pm) at an ambient temperature of 21–22 °C with 50–60% relative humidity. All of the mice were allowed to adapt to the facility conditions for one week before the experiments began. The mice were randomly divided into four groups, a vehicle control group (*n* = 5) and three drug-treatment groups with doses of 5 mg/kg (*n* = 5), 10 mg/kg (*n* = 5), and 20 mg/kg (*n* = 6). ATRA (Sigma-Aldrich, St Louis, MO, USA) was suspended in ethanol and diluted in corn oil [47]. All of the groups were injected in the abdominal cavity between 18:00 and 20:00 daily for 12 days with a volume of 2.5 mL/kg body weight. Then we started behavioral testing on day 13 accompanied with injection during the behavioral tests [37,48]. Behavioral testing was conducted between 9:00 and 15:00. A diagram of the behavioral testing procedure is shown in Figure 6. All of the animal experiments were performed in accordance with the Guide for the Care and Use of Laboratory Animals of the University of Science and Technology of China and were approved by the Animal Care and Use Committee at the University of Science and Technology of China (number USTCACUC1901015, approval date April 2019). 

### 4.2. Behavioral Tests

#### 4.2.1. Open-Field Test (OFT)

The open-field test (OFT) was used to analyze spontaneous exploratory activity and evaluate the level of anxiety in mice. The open-field apparatus included a white floor (50 cm × 50 cm) and 25-cm-high walls. The floor was divided into 16 equal squares with black lines. Four central squares were considered the central area. Each mouse was gently placed in the corner facing the wall, separately and was left free to explore the unfamiliar open field for 5 min. The room was kept quiet during the entire test, and the apparatus was cleaned with 75% ethanol after each trial. The time, distance and velocity traveled in the central area were recorded [49]. The data were analyzed using a video camera and processed with EthoVision (Noldus, Wageningen, The Netherlands). 

#### 4.2.2. Elevated-Plus Maze Test (EPM)

The elevated-plus maze test (EPM) was used to evaluate anxiety-like behavior. The apparatus, made of white Plexiglas^®^, was elevated 50 cm above the floor and consisted of two opposite open arms (30 cm × 6 cm, without walls) and two opposite closed arms (30 cm × 6 cm) with walls (15-cm height). The apparatus had a central area (6 cm × 6 cm). Each mouse was placed in the central arena of the maze facing an open arm and allowed to explore for 5 min. The room was kept quiet during the entire test, and the apparatus was cleaned with 75% ethanol after each trial. The time and distance traveled in each type of arm and the number of entries into an open arm were recorded [49]. The percentage (%) of time spent in an open arm was calculated. The data were analyzed using a video camera and processed with EthoVision (Noldus). 

#### 4.2.3. Forced Swimming Test (FST)

The forced swimming test (FST) was used to evaluate depression-like behavior. The mice were placed into a circular bucket (height, 25 cm; diameter, 10 cm) containing water (15-cm depth) maintained at 22 ± 2 °C. Mice were placed in the container with their back to the wall and with the front paws touching the water. Each mouse was allowed to swim freely for 6 min. After each session, the mouse was dried with a towel and returned to its home cage. The room was kept quiet during the entire test. The mobility time was recorded. Each mouse was judged to be moving when swimming, struggling, or climbing. Floating motionless in the water and making only those movements necessary to keep its head above water were judged to be immobility [50]. Decreased duration of mobility during the FST was taken as a measure of depression-like behavior.

#### 4.2.4. Sucrose Consumption Test (SPT)

The sucrose consumption test (SPT) was described previously [38,51]. Mice were housed individually and were trained to drink from two bottles containing 1% (wt/vol) sucrose solution for 24 h. Animals were then weighed and placed back in a cage, with free choice between two bottles, one containing 1% sucrose solution and one tap water, for 24 h. To prevent left/right preference, the position of the bottles was switched after a 12-h test. No previous food or water deprivation was applied before the test. The weight of water and sucrose consumed was measured after 24 h. The percentage of sucrose solution from the total liquid consumed was calculated, as a measure of anhedonia.

### 4.3. Tissue Preparation

After the behavioral tests, whole brains were collected immediately following killing by decapitation. Hippocampus tissue was dissected and quickly frozen in liquid nitrogen, and then stored at −80 °C for analysis by quantitative real-time polymerase chain reaction (qPCR). The member of different groups was collected randomly.

### 4.4. RNA Isolation and Quantitative Real-Time Polymerase Chain Reaction

For RNA isolation from brain tissue, we used the TRIzol method (Invitrogen; Thermo Fisher Scientific, Waltham, MA, USA) [52]. The total RNA was quantified by a One Drop OD-1000 spectrophotometer (Nanjing Wuyi Technology Co., Ltd., Nanjing, China) and an equal quantity of RNA (500 ng) was reverse transcribed (TaKaRa, Kyoto, Japan) to synthesize cDNA. The relative amount of target gene was calculated using the 2^−∆∆*C*t^ method [53] with quantitative real-time polymerase chain reaction (qPCR) on a StepOne platform (Applied Biosystems, Foster City, CA, USA). qPCR was performed in a total 20 μL reaction volume containing 10 μL SYBR Green Master mix (TaKaRa) for 40 cycles (15 s at 95 °C and 1 min at 60 °C). The mRNA level of β-actin was used as an internal control. 

The mice primers used are shown as follow: *β-actin*: F: 5’-ACTCCTATGTGGGTGACGAG-3’, R: 5’-CATCTTTTCACGGTTGGCCTTAG-3’; *RARα*: F: 5’-TTCTTTCCCCCTATGCTGGGT-3’, R: 5’-GGGAGGGCTGGGTACTATCTC-3’; *RARβ*: F: 5’-GCAGTGCGTGGACACATGA-3’, R: 5’-GGCAGGGAGAGTCCTCTGAT-3’; *RARγ*: F: 5’-GGAGCAGGCTTCCCATTCG-3’, R: 5’-CATGGCTTATAGACCCGAGGA-3’; *DLG2*: F: 5’-CTGTCACGAGGCAGGAAATAAA-3’, R: 5’-CGACTTCGTAGTCACGCTTTG-3’; *SynDIG1*: F: 5’-CATGCTGCTTCAGTTGCCAG-3’, R: 5’-AGTCCAAACATCACCATTCATCA-3’.

### 4.5. Statistical Analysis

Data analyses were performed using IBM SPSS version 19.0 (IBM Corp., Armonk, NY, USA). Values are expressed as mean ± standard error of the mean (SEM). The differences between groups were tested by one-way analysis of variance (ANOVA), followed by an LSD post-hoc test. Correlation analysis was performed using a Pearson’s correlation test. A *p*-value < 0.05 was considered to be statistically significant.

## 5. Conclusions

Our results show that administration of ATRA induced anxiety- and depression-like behavior in young mice, accompanied by a decreased expression of the synaptic gene *DLG2* and an increased expression of *SynDIG1*. *DLG2* mRNA levels were correlated with anxiety-like behavior and positively correlated with *RARγ*. *SynDIG1* mRNA levels was correlated with depression-like behavior and negatively correlated with *RARγ*. Our study findings reveal possible novel links between retinoid signaling and depression. 

## Supplementary Materials

Supplementary materials can be found at https://www.mdpi.com/1422-0067/21/8/2677/s1.

## Abbreviations

AMPAR	α-amino-3-hydroxy-5-methylisoxazole-4-propionic acid subtype glutamate receptors
ATRA	all-trans retinoic acid
CNS	central nervous system
DLG2	discs large homolog 2
EPM	elevated-plus maze
FST	forced swimming test
HPA	hypothalamic-pituitary-adrenal axis
LSD	least significant difference
NMDAR	N-methyl-D-aspartate receptor
OFT	open-field test
RARα	retinoic acid receptor α
RARβ	retinoic acid receptor β
RARγ	retinoic acid receptor γ
SPT	sucrose preference test
SynDIG1	synapse differentiation-inducing gene protein 1
