# Supplementary material for: All-trans Retinoic Acid-induced Abnormal Hippocampal Expression of Synaptic Genes SynDIG1 and DLG2 is Correlated with Anxiety or Depression-Like Behavior in Mice
Source: Int J Mol Sci. 2020 Apr 11;21(8):2677. doi: 10.3390/ijms21082677 (PMC7215843; doi:10.3390/ijms21082677)
Supplement: Supplementary file 1 [file ijms-21-02677-s001.pdf]

International Journal of *Molecular Sciences*

Table S1. Genes that are altered by ATRA.

Gene Symbol	Fold Change	Regulation	Mean ATRA	Mean Control	Gene Bank Accession
CRYGC	106.99	up	11.95	5.21	NM_020989
GNG8	21.99	up	12.10	7.64	NM_033258
KRT86	21.79	up	9.88	5.43	NM_002284
HTR2B	15.070	up	11.64	7.73	NM_000867
DLG2	14.21	up	9.85	6.01	NM_001364
SynDIG1	0.18	down	4.23	6.67	NM_024893
CHST15	0.17	down	6.24	8.76	NM_015892
MT1G	0.16	down	5.11	7.71	NM_005950
NELL1	0.15	down	4.39	7.09	NM_006157
IGSF21	0.11	down	4.86	8.05	NM_032880
ADAMTS17	0.065	down	3.85	7.79	NM_139057

Microarray experiment and data analysis were performed in Shanghai Biotechnology Corporation. “Gene Symbol” column refers to the genes abbreviation. “Fold change” column indicates the changes of expression levels between the ATRA group and control group. “Regulation” column refers to the variation trends of genes expression levels regulated by ATRA compared to the vehicle. “up” refers to the up-regulation of genes expression by ATRA. “down” refers to the down-regulation of genes expression by ATRA. “Mean ATRA” columns refer to the average expression levels of samples treated with ATRA. “Mean Control” columns refer to the average expression levels of samples treated with DMSO. “Gene bank Accession” column refers to the accession number of each gene in gene bank. The human neuroblastoma cell line BE2(c) (purchased from ATCC) were grown in DMEM /F12 (DF; Sigma, St. Louis, Missouri) medium supplied with 10% fetal bovine serum (FBS; Gibco, Grand Island, New York). The cell lines were cultured in 5% CO2 humidified atmosphere maintained at 37 °C. The cells were treated with 1 μM ATRA (Sigma; soluble in DMSO) or the same volume of solvent, DMSO, for 24 hours. Before drug treatment, the medium was replaced with a phenol red-free medium plus 10% dextran coated charcoal–treated FBS. Cells were collected with TRIzol 24 hours later after ATRA administration. Abbreviation: ATRA, All-trans retinoic acid; CRYGC, crystallin, gamma C; DMSO, dimethyl Sulfoxide; GNG8, guanine nucleotide binding protein (G protein), gamma 8; KRT86, keratin 86; HTR2B, 5-hydroxytryptamine (serotonin) receptor 2B, G protein-coupled; DLG2, discs, large homolog 2 (Drosophila); SynDIG1, synapse differentiation inducing 1; CHST15, carbohydrate (N-acetylgalactosamine 4-sulfate 6-O) sulfotransferase 15; MT1G, metallothionein 1G; NELL1, NEL-like 1 (chicken); IGSF21, immunoglobin superfamily, member 21; ADAMTS17, ADAM metallopeptidase with thrombospondin type 1 motif, 17.

Table S2. Correlation of the DLG2/SynDIG1 mRNA levels in the hippocampus with behavior.

<table>
<tr><th>Classification</th><th>Behavior</th><th>Parameter</th><th></th><th>DLG2</th><th>SynDIG1</th></tr>
<tr><td rowspan="8">Anxiety-associated</td><td rowspan="4">Open-field test</td><td rowspan="2">Duration in center</td><td>r</td><td>0.5420*</td><td>−0.3763</td></tr>
<tr><td>p</td><td>0.0111</td><td>0.0927</td></tr>
<tr><td rowspan="2">Distance in center</td><td>r</td><td>0.5128*</td><td>−0.3571</td></tr>
<tr><td>p</td><td>0.0174</td><td>0.1120</td></tr>
<tr><td rowspan="4">Elevated-plus maze test</td><td rowspan="2">Duration in open arms</td><td>r</td><td>0.1289</td><td>−0.3431</td></tr>
<tr><td>p</td><td>0.5778</td><td>0.1278</td></tr>
<tr><td rowspan="2">Distance in open arms</td><td>r</td><td>0.2455</td><td>−0.2996</td></tr>
<tr><td>p</td><td>0.2834</td><td>0.1871</td></tr>
<tr><td rowspan="2">Depression-associated</td><td rowspan="2">Forced swimming test</td><td rowspan="2">Mobility time</td><td>r</td><td>0.06421</td><td>−0.5861**</td></tr>
<tr><td>p</td><td>0.7821</td><td>0.0052</td></tr>
</table>

The correlation analysis was performed by Pearson's correlation test (n = 21). Asterisks (*) show the significance of the correlation. *$p < 0.05$, **$p < 0.01$.
